# Characterization of CHARK, an unusual cytokinin receptor of rice

**DOI:** 10.1038/s41598-020-80223-2

**Published:** 2021-01-18

**Authors:** Mhyeddeen Halawa, Anne Cortleven, Thomas Schmülling, Alexander Heyl

**Affiliations:** 1 Institute of Biology/Applied Genetics, Dahlem Centre of Plant Science, Freie Universität Berlin, Albrecht-Thaer-Weg 6, 14195 Berlin, Germany; 2 Biology Department, Adelphi University, 1 South Avenue, Garden City, NY 11530-0701 USA

**Keywords:** Molecular biology, Plant sciences

## Abstract

The signal transduction of the plant hormone cytokinin is mediated by a His-to-Asp phosphorelay. The canonical cytokinin receptor consists of an extra cytoplasmic hormone binding domain named cyclase/histidine kinase associated sensory extracellular (CHASE) and cytoplasmic histidine kinase and receiver domains. In addition to classical cytokinin receptors, a different type receptor—named CHASE domain receptor serine/threonine kinase (CHARK)—is also present in rice. It contains the same ligand binding domain as other cytokinin receptors but has a predicted Ser/Thr—instead of a His-kinase domain. Bioinformatic analysis indicates that CHARK is a retrogene and a product of *trans*-splicing. Here, we analyzed whether CHARK can function as a *bona fide* cytokinin receptor. A biochemical assay demonstrated its ability to bind cytokinin. Transient expression of CHARK in protoplasts increased their response to cytokinin. Expression of CHARK in an Arabidopsis receptor double mutant complemented its growth defects and restored the ability to activate cytokinin response genes, clearly demonstrating that CHARK functions as a cytokinin receptor. We propose that the *CHARK* gene presents an evolutionary novelty in the cytokinin signaling system.

## Introduction

Cellular signaling systems are crucial components of life, as they allow an organism to perceive and react to changes in its environment or to coordinate developmental processes. Therefore, it is not surprising that much attention has been given to characterizing and understanding cellular signaling pathways across all kingdoms. One aspect of this research is the origin and evolution of signaling pathways. The advent of the genomic era via the application of high throughput sequencing and the dramatic increase of sequenced genomes has greatly facilitated the investigation of the evolution of whole pathways.

One of the fascinating themes in the evolution of signaling pathways is the reuse of existing components rather than their de novo synthesis^1–5^. One example for reuse of existing components is the signaling pathway of the phytohormone cytokinin. Cytokinins are adenine derivatives that control many aspects of plant development and the responses to changes in the abiotic and the biotic environment^6–8^. The cytokinin signaling pathway is a multi-step variant of the bacterial two-component signaling system and is believed to have been introduced to the plant lineage via the endosymbiosis of cyanobacteria^9,10^. Cytokinin is perceived by membrane-bound hybrid histidine kinases, which then relay the signal to downstream components in the cytosol^11,12^. These cytokinin receptors bind cytokinin by a cyclase/histidine-kinase associated sensory extracellular (CHASE) domain^13^. The binding is thought to be transmitted to the cytoplasmic part of the receptors by conformational changes, triggering the autophosphorylation of the receptor at the histidine kinase domain^14–16^. At least in Arabidopsis, other closely related histidine kinases do not seem to play a role in cytokinin signaling^17^. All CHASE domains identified in land plants are always found in combination with histidine kinase domains^18,19^. The only exception is a CHASE domain receptor serine/threonine kinase (CHARK), which combines the CHASE domain not with a His kinase, but with a predicted Ser/Thr kinase domain^19,20^. Its Ser/Thr kinase domain has been reported to be most closely related to that of a lectin receptor like kinase of the Concavalin A class^20^, while its CHASE domain is most closely related to the rice cytokinin receptor OsHK3^19^. *CHARK* is mainly expressed in roots and spikelets, but low amounts of transcript can be also found in other types of tissue^20–22^.

Because of the unusual domain composition of CHARK, we wondered if this protein might represent a novel side branch of the well-characterized cytokinin signaling pathway and analyzed CHARK for its ability to function in cytokinin signaling. It was found that CHARK binds cytokinin in a heterologous assay. More importantly, however, CHARK was shown to complement the *ahk2 ahk3* cytokinin receptor mutant of Arabidopsis, on both the morphological and the molecular level. Thus, the characterization of CHARK as a cytokinin receptor might provide insights into the early steps of a path of receptor evolution in general.

## Methods

### Bioinformatic analysis

The domain structure of CHARK was determined using the PFAM and the TMHMM programs^23,24^. In order to infer the phylogenetically most closely related domains in the rice genome, BLAST searches^25^ were conducted using the default settings. For the CHASE domain all found proteins were used and for the Ser/Thr kinase domain the top eight hits were included in the subsequent analysis. Alignments and tree calculations were done using the MEGA7 program suit^26^. The sequences were aligned using the MUSCLE program and a Maximum Likelihood tree was calculated for the two sets of proteins respectively.

### Cytokinin binding assay

The cytokinin binding assay was performed as described before^27^. The cytokinin receptor *Arabidopsis* histidine kinase AHK4 and the putative cytokinin receptor CHARK of *Oryza sativa* were cloned into the pDEST15 vector (Invitrogen, Karlsruhe, Germany) and expressed in the *E. coli* strain KMI002^27^. The empty vector pDEST15 was used as a negative control. Tritium-labeled *trans*-[^3^H]zeatin (592 GBq/mmol) was obtained from the Isotope Laboratory of the Institute of Experimental Botany (Prague, Czech Republic). The protein expression was confirmed by western blot.

### Plant material and growth conditions

*Arabidopsis thaliana* Col-0 (wild type) and the *Arabidopsis thaliana* receptor mutant line *ahk2-2 ahk3-3* were used for the plant complementation analyzes^28^. The *Arabidopsis thaliana* receptor mutant line *ahk2-5 ahk3-7*^29^ was used for the prototoplast *trans*-activation assay (PTA). For the sterile in vitro culture of *Arabidopsis thaliana*, MS medium (4.2 g/l MS salts, 0.1 g myo-inositol, 0.5 g/l MES, 10 g/l sucrose, pH 5.7 with KOH and 9 g/l agar^30^ was used. 10 μg ml^−1^ phosphinotricin (PPT) was added to the medium to select the transformed *Arabidopsis* plants. Seeds were surface sterilized with 1.2% sodium hypochlorite solution. After sowing and vernalisation, they were grown in a growth cabinet (AR66-L, Percival Scientific, Perry, USA) under long-day conditions (16 h:8 h—L:D; 75 µmol m^−2^ s^−1^). For the non-sterile plant cultures, *Arabidopsis* seeds were sown on soil (2:2:1 P-soil:T-soil:perlite) in the greenhouse at 22 °C under long-day conditions (16 h:8 h—L:D) or the plants preselected on medium were picked on soil about 14 d after germination. For reverse transcription quantitative PCR (RT-qPCR) analysis, seeds were surface-sterilized, stratified at 4 °C for 2 d and then grown for 2 weeks on half MS liquid medium (0.1% sucrose) in a growth cabinet (Percival AR66L; Percival Scientific, Perry, USA) at 22 °C and a light intensity of 150 µmol m^−2^ s^−1^. Cytokinin treatment was with 1 µM BA for 1 h.

### Protoplast *trans*-activation assay (PTA)

Protoplast isolation and transformation were carried out according to the method described earlier^31^. Four- to five-weeks-old rosette leaves were used for the isolation of mesophyll protoplasts. The transformation of protoplasts was mediated by a 40% polyethylene glycol (PEG) solution. The protoplasts were incubated overnight with 500 nM *trans*-zeatin (*t*Z) for cytokinin treatment. A 9 µg aliquot of the *ARR6:GUS* reporter plasmid, 14 µg of the effector plasmid carrying the *35S:ARR2* gene and 14 µg of the cytokinin receptor gene under control of the 35S promoter (pB2GW7 vector) was used for the transactivation assays. The empty pBT10-GUS vector was used as a negative control. Then 3 µg of a plasmid harboring the *35S:NAN* construct^32^ was added for normalization. Both GUS and NAN enzyme assays were performed according to^32^ . The ratios of GUS and NAN activities were calculated as relative GUS/NAN activity units.

### Complementation analyzes in *Arabidopsis thaliana*

To investigate the activity of CHARK in plants, complementation analyzes were performed in the *ahk2-2 ahk3-3 Arabidopsis thaliana* receptor mutant line^28^. The *CHARK* gene was expressed under the control of the *AHK2* promoter and with a C-terminal myc-tag using the pB7m34GW vector^33^. After the segregation analyses, three independent homozygous lines of *pAHK2-CHARK* (*pAHK2-CHARK 22*, *pAHK2-CHARK 24* and *pAHK2-CHARK 25*) were obtained and the expression of *CHARK* in these lines was confirmed by western blot. The complementation of the *ahk2-2 ahk3-*3 mutant in those three lines was determined by three assays (rosette diameter, shoot length, and root growth). Rosette diameter and shoot height were measured at 25 and 34 days after germination (dag), respectively. The experiment was repeated twice (n ≥ 24). For the lateral root assay, seedlings of all tested lines were grown on vertical plates containing half-strength MS medium supplemented with 0, 10, 25, 50, or 100 nM iP [N^6^-(Δ^2^-Isopentenyl)adenine] and lateral roots were counted.

### RNA isolation and reverse transcription quantitative PCR

RT-qPCR was performed with RNA of two-week old wild-type or homozygous transgenic seedlings which were treated with 1 µM BA dissolved in KOH for 1 h or with a mock treatment (solvent control). Seedlings were grown in half MS liquid medium containing 0.1% sucrose. Total RNA was extracted from entire two-week old seedlings using the NucleoSpin RNA plant kit (Macherey and Nagel, Düren, Germany) as described in the user’s manual. After a DNAse step (Fermentas, Life Technologies, Darmstadt, Germany), equal amounts of RNA (1 µg RNA) were used in a 20 µl SuperScript III Reverse Transcriptase reaction. First strand cDNA synthesis was primed with a combination of oligo(dT)-primers and random hexamers. Primer pairs were designed using Primer 3 Software (http://www.genome.wi.mit.edu/cgibin/primer/primer3.cgi) under the following conditions: optimum Tm at 60 °C, GC content between 20 and 80%, 150 bp maximum length. Primers used for reference genes and genes of interest are listed in Table S1. Real-time PCR using SYBR Green I technology was performed on an CFX-96-Real-Time System (C1000 Touch Thermal Cycler) (Biorad, Munich, Germany) and the following cycling conditions (15 min 95 °C, 40 cycles of 15 s at 95 °C, and 15 s at 55 °C and 10 s at 72 °C), followed by the generation of a dissociation curve to check for specificity of the amplification. Reactions contained 0.01 U/µl Immolase (Bioline, Luckenwalde, Germany), SYBR Green (Applied Biosystems, Life Technologies, Darmstadt, Germany), 2 µM MgCl_2_, 0.1 µM dNTPs and 300 nM of a gene specific forward and reverse primer and 2 µl of a 1:10 diluted cDNA in a 20 µl reaction. Gene expression data were normalized against two different nuclear-encoded reference genes (*EF1A* and *UBC21*) according to^34^ and presented relative to the WT control treatment.

## Results

### CHARK sequence reveals unusual features

**Figure 1:**
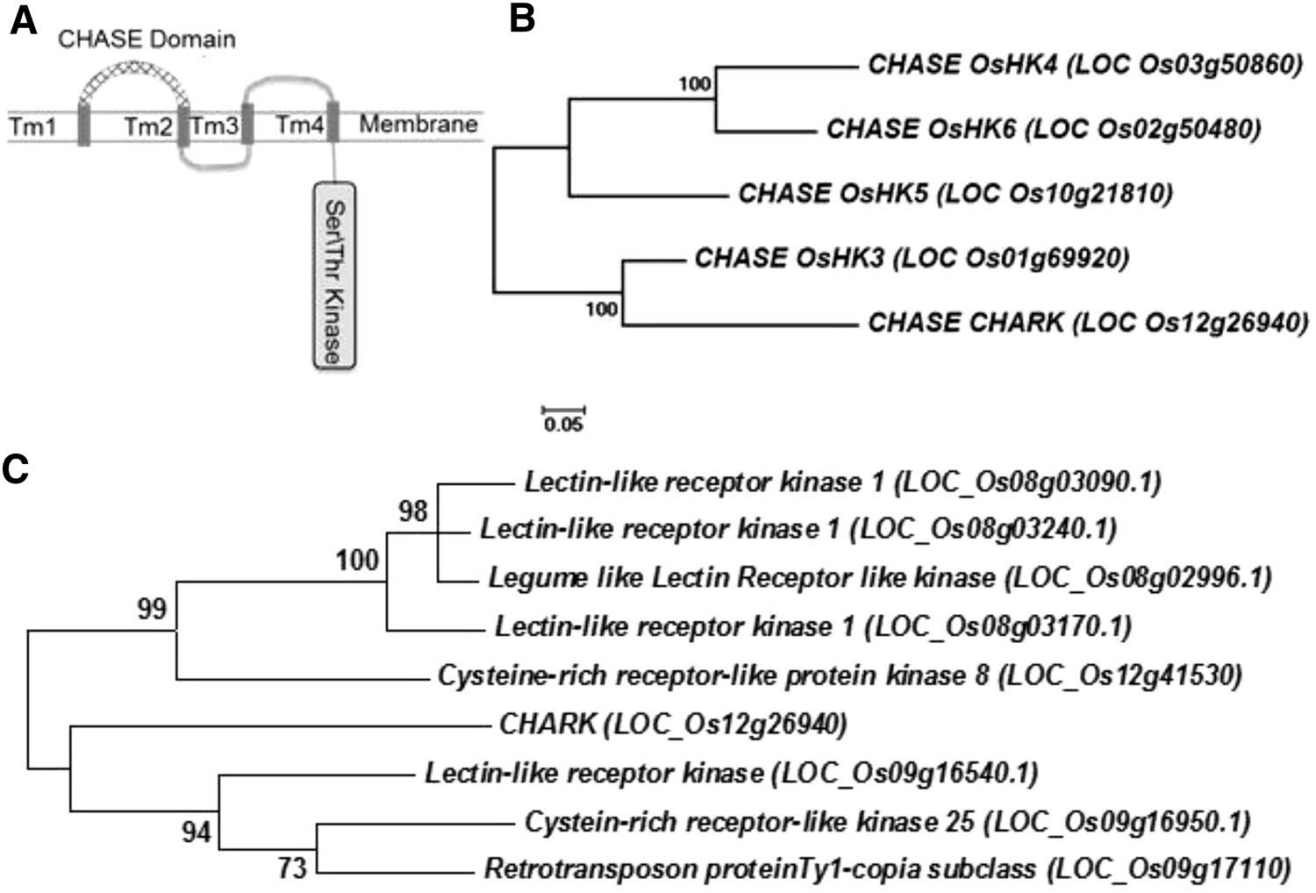
Domain structure and phylogenetic relations of CHARK. (**A**) Domain structure of CHARK. The domain structure of CHARK was determined using the PFAM and the TMHMM programs^23,60^. (**B**,**C**) Phylogenetic relations of the CHASE and Ser/Thr kinase domains. The phylogenetic most closely related sequences to CHARK were determined by using the sequence of both domains, CHASE and Ser/Thr kinase, respectively, as query in BLAST searches to identify the most closely related sequences in rice. The resulting sequences were aligned using MUSCLE and Maximum Likelihood trees were calculated, respectively, using the MEGA7 program^26^. *Tm* single membrane-spanning helix.

Sequence analysis revealed that the CHARK sequence from *Oryza sativa *cv.* japonica* obtained from the Gramene database^35^ is 948 aa long and contains in addition to the CHASE domain (70–279 aa), known to be responsible for the binding of cytokinin, a protein kinase domain, which contains a Ser/Thr kinase signature (641–944 aa) (Fig. 1A) (PFAM^24^). The CHASE domain of CHARK is most closely related to that of OsHK3, a known cytokinin receptor from rice (Fig. 1B)^36^. The Ser/Thr domain of CHARK was most closely related to the kinase domain of LOC_Os09g16540, an L-type lectin receptor like kinase (Fig. 1C)^37^.

### Phylogenetic analysis of CHARK

**Figure 2:**
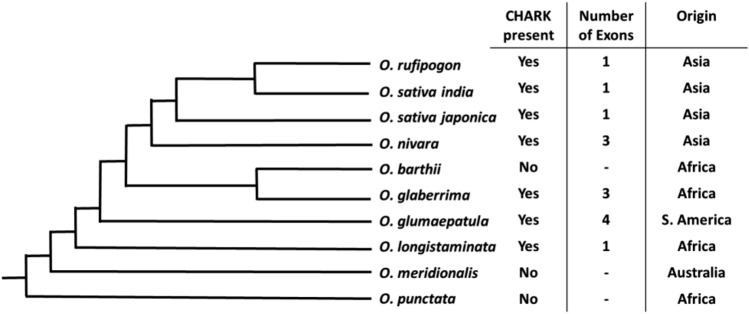
Phylogeny of CHARK in different rice species. The homologues for CHARK were searched for in ten rice species using the Gramene database^35^. The exon/intron structure was determined and the continent of origin of the species was identified. These findings were visualized by plotting them on a phylogeny of the different rice species which was published previously^38^.

To gather more information about the evolutionary origin of CHARK and its distribution across the different plant species related to rice, the Gramene database was queried^35^. Homologous genes were found only in seven closely related accessions of the eleven rice species for which sequence data were available and not in any other plant species (Fig. 2). A more in-depth analysis of the *CHARK* gene sequences showed that in those species that do encode for CHARK in their genome, only one copy of the gene is present. Mapping the presence of CHARK on the phylogenetic tree for *O. sativa* and its relatives^38^ revealed the first presence of CHARK in *O. longistaminata*, a rice species from Africa (Fig. 2). All later diverging species did encode for CHARK in their genome with the exception of *O. barthii*, which might have lost the *CHARK* gene secondarily. Interestingly, the gene is intronless in the most ancient species (*O. longistaminata*) and the three latest divergent species, as reported before^39^, while the *CHARK* genes of the other rice accessions contain 2–3 introns. Geographically, *CHARK* sequences can be found in rice species from all continents of its natural distribution, with the exception of Australia (Fig. 2).

### CHARK displays properties of a classical cytokinin receptor

**Figure 3:**
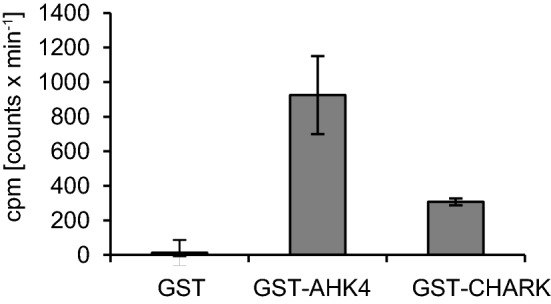
Specific cytokinin binding activity of CHARK in an in vivo binding assay. AHK4 and CHARK were expressed as GST fusion proteins in *E. coli* strain KMI002 and bacterial cells were assayed for specific *trans*-[^3^H]zeatin binding^27^. The empty pDEST15 vector was used as a negative control. The assay was performed with three independent bacterial colonies for each construct and the binding activity was measured twice for each colony. Error bars indicate the standard deviation (*n* = 3). The protein expression was confirmed by western blot analysis with GST antibody (Supplemental Fig. S1).

There are two basic functions any receptor has to accomplish in order to be functional: to bind its ligand and to convert this ligand binding into a cellular signal. To test if CHARK binds to its ligand, its open reading frame (ORF) was cloned and expressed in *E. coli* to be used in a bacterial cytokinin binding assay^27^. In this assay, the best-characterized cytokinin receptor, Arabidopsis Histidine Kinase 4 (AHK4), served as a positive control, and the empty vector as a negative control. The ORFs were expressed in *E. coli* as GST-fusion proteins. Expression of the different proteins was confirmed by western blot (Supplemental Fig. S1). Bacteria expressing the different constructs were incubated with ^3^H-labelled cytokinin (*trans*-zeatin) and after washing, the bound cytokinin was measured. AHK4 showed strong cytokinin binding, while the binding detected for GST alone was negligible. The cytokinin binding detected for CHARK was clearly above background, but not as strong as for AHK4 (Fig. 3).

**Figure 4:**
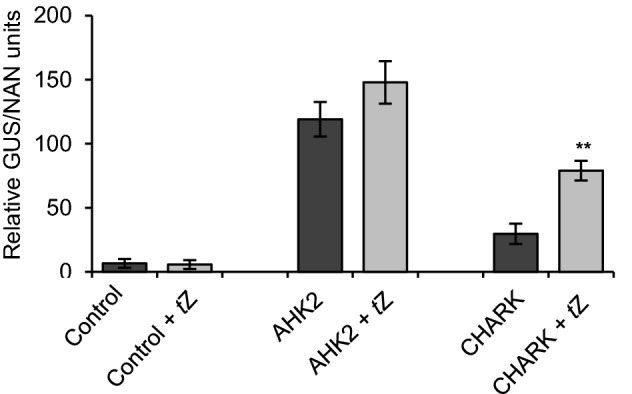
Cytokinin receptor assay in *ahk2 ahk3* receptor mutant protoplasts. Mesophyll protoplasts of *A. thaliana* receptor mutant line *ahk2-5 ahk3-7* were co-transfected with the *pARR6:GUS* reporter gene, *35S:ARR2* and *35S:CHARK* or *35S:AHK2* (positive control) or the empty vector (negative control)^36^. For cytokinin treatment, the protoplasts were incubated overnight with 500 nM *trans*-zeatin (*t*Z). Data represent mean values of three biological replicates ± SD. Student’s *t*-test was used to calculate the significance of differences between untreated samples and cytokinin-treated samples. **P < 0. 01.

The second prerequisite for the function of a receptor, the conversion of the binding into a cellular signal, was first tested using a transient *in planta* complementation assay with Arabidopsis protoplasts^36^. Protoplasts of the *ahk2 ahk3* mutant, a double knockout mutant for two of the three cytokinin receptors from Arabidopsis, *AHK2* and *AHK3*^29^, were transformed with *CHARK* and *AHK2* under the control of the 35S promoter. The empty expression vector served as a negative control. The level of compensation of the mutations was measured with the help of a *GUS* reporter gene under the control of the cytokinin-responsive *ARR6* promoter. As reported before, the transformation of a functional cytokinin receptor reestablished the ability to sense cytokinin and transduce its signal, while the empty vector control displayed only background levels of GUS activity^36^. Expression of CHARK activated the reporter, although less strongly than AHK2, indicating activation of the cytokinin signaling pathway (Fig. 4). The signaling output of protoplasts expressing *CHARK* nearly doubled upon cytokinin treatment, an increase that was significantly stronger than that detected for AHK2. These results suggest that the CHARK protein is able to sense cytokinin and transduce its binding into a cellular signal.

In summary, these results demonstrated that CHARK can bind cytokinin and can function as a cytokinin receptor—at least in protoplasts.

### CHARK complements an Arabidopsis cytokinin receptor mutant

**Figure 5:**
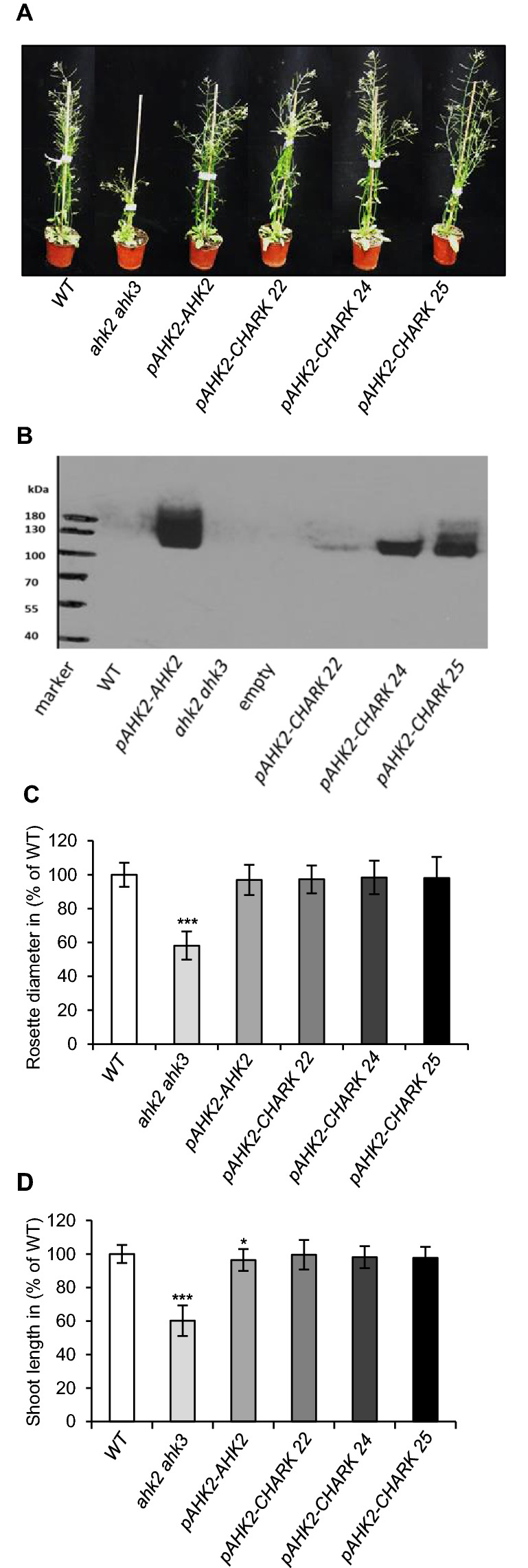
*CHARK* complements functionally the *A. thaliana* receptor mutant line *ahk2 ahk3*. (**A**) Complementation of the *ahk2-2 ahk3*-*3* mutant by *pAHK2-AHK2*^28^ (positive control) or *pAHK2-CHARK*. Three independent homozygous lines containing *pAHK2-CHARK* were tested. Plants shown are 25 days old. (**B**) The expression of the AHK2-myc and CHARK-myc fusion proteins was detected by protein blot analysis using a myc antibody. (**C**,**D**) Rosette diameter and shoot length of WT, *ahk2-2 ahk3*-3, *pAHK2-AHK2* and the three lines expressing *CHARK*. The experiments were repeated twice. Data represent mean values ± SD (*n* ≥ 24). The significance of differences compared to WT were calculated using Student’s t-test. *P < 0.05; ***P < 0.001.

In order to clearly demonstrate its function as cytokinin receptor, we tested whether *CHARK* can genetically complement the Arabidopsis *ahk2 ahk3* double mutant. The *ahk2 ahk3* double mutant was chosen as it has a distinct shoot phenotype with a smaller rosette and a reduced plant height^29^. The *ahk2 ahk3 ahk4* triple mutant is extremely small and thus impractical to work with^29^. *CHARK* was expressed with a myc-tag under the control of the *AHK2* promoter, which proved to be more effective in previous complementation experiments and transformed into the double mutant^28^. Of the ten independent lines obtained, three (pAHK2-CHARK 22, pAHK2-CHARK 24, pAHK2-CHARK 25) were chosen for a more detailed analysis (Fig. 5A). The *ahk2 ahk3* mutant line transformed with *pAHK2-AHK2* was used as positive control for the complementation. The expression of the transgenes was confirmed by a western blot analysis using an anti-myc antibody (Fig. 5B). Two morphological features, plant height and rosette diameter, were quantified in the transgenic plant lines and compared to the wild type (Fig. 5C,D). Both *AHK2* and *CHARK* expression restored the smaller rosette size of the *ahk2 ahk3* mutant to the wild-type size (Fig. 5C). The same was true for the plant height. While the *ahk2 ahk3* plants reached only about 60% of the height of the wild type, expressing either *AHK2* or *CHARK* in those plants resulted in plant heights comparable to those of the wild type (Fig. 5D). These data demonstrated that CHARK can functionally complement a cytokinin receptor mutant *in planta*.

### CHARK restores the cytokinin response in the *ahk2 ahk3* mutant

**Figure 6:**
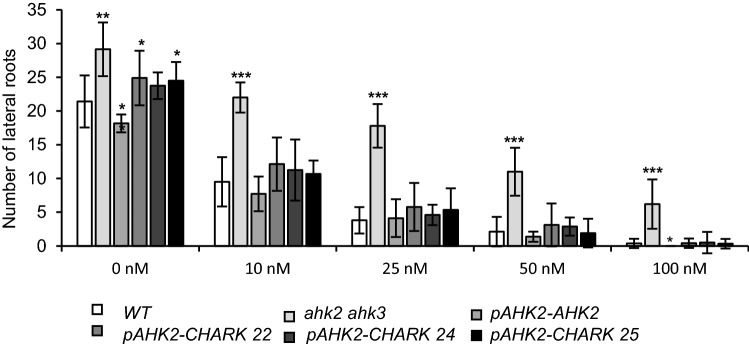
CHARK mediates suppression of lateral root formation by cytokinin. The number of lateral roots of seedlings grown on medium supplemented with 0, 10, 25, 50, or 100 nM iP was counted 12 days after germination. The experiment was repeated three times. Data represent mean values ± SD (*n* = 10). The significance of differences compared to WT was calculated using Student’s t-test. *P < 0.05; **P < 0.01; ***P < 0.001.

Next, we tested whether CHARK is able to restore the cytokinin response in the *ahk2 ahk3* mutant as well. One of the prominent effects of cytokinin on plant development is the repression of root growth^40^. Thus, the lateral root assay was used to test the ability of CHARK to restore cytokinin responses. In the wild type, 10 nM of isopentenyl adenine (iP) reduced the number of lateral roots by more than 50% as compared to the untreated control (Fig. 6). Higher concentrations of iP further reduced the number of lateral roots and at 100 nM iP almost no lateral roots were formed. In contrast, the *ahk2 ahk3* mutant displayed hyposensitivity to cytokinin as the number of lateral roots decreased much less at increasing concentrations of the hormone. 50 nM iP were needed to reach a 50% reduction and even at the highest tested cytokinin concentration (100 nM iP), the double receptor mutant still had some lateral roots (Fig. 6). In the lines expressing either *AHK2* or *CHARK* in the double mutant background, the level of lateral root formation was comparable to that of the wild type across the different cytokinin treatments, which confirms the complementation of the *ahk2 ahk3* mutation.

Together, the lateral root assay showed that CHARK can, besides morphologically complementing the cytokinin receptor mutants, also restore their response to cytokinin treatment.

**Figure 7:**
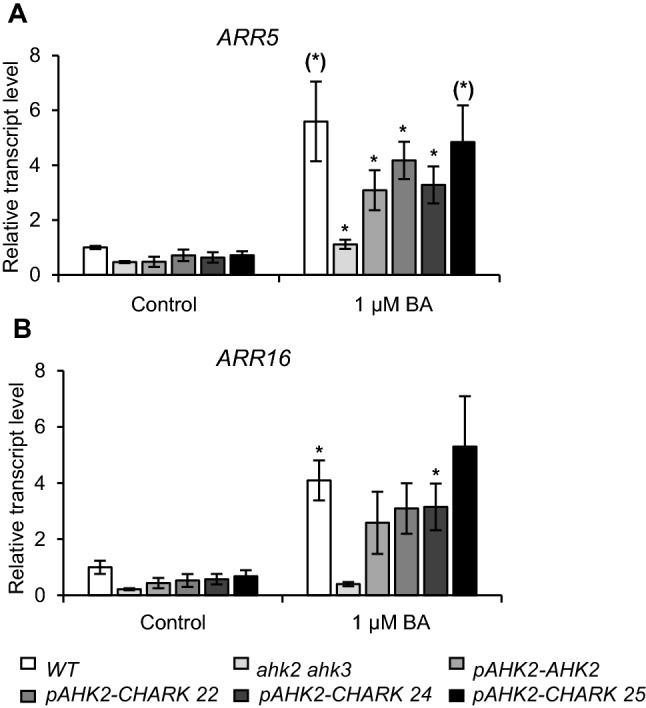
CHARK mediates the activation of cytokinin response genes in *A. thaliana.* Induction of (**A**) *ARR5* and (**B**) *ARR16* expression by cytokinin in WT, *ahk2-2 ahk3-3*, *pAHK2-AHK2*, and three independent lines expressing *pAHK2-CHARK* in the *ahk2 ahk3* mutant background. Two-weeks old seedlings were treated for 1 h with 1 µM BA. Expression levels of *ARR5* and *ARR16* were analyzed by qRT-PCR. Data represent mean values (± SE) (n = 4). Student’s t-test was used to calculate the significance of differences between cytokinin-treated samples and untreated samples. *P ≤ 0.05; ^(^*^)^*P* = 0.051.

In order to test if CHARK also activates the cytokinin response on the molecular level, the expression of two cytokinin response genes were investigated in the different plant lines. Both *ARR5* and *ARR16* belong to the type-A response regulator genes (*ARRs*) and represent primary cytokinin response genes^41,42^. Seedlings were treated for 1 h with 1 μM of the cytokinin BA (6-benzyladenine) or a solvent control, before transcript levels were measured by real-time qRT-PCR. In the wild type, the expression levels of *ARR5* and *ARR16* was 5.5- and 4-fold higher after cytokinin treatment than in the control, respectively (Fig. 7). In contrast, in the *ahk2 ahk3* double mutant, only the transcript level of *ARR5* increased slightly after BA-treatment, thus indicating a strongly diminished cytokinin response. The expression of *AHK2* in the double mutant resulted in the partial restoration of the cytokinin induction of the two *ARR* genes (Fig. 7). Similarly, in the three different *ahk2 ahk3* lines expressing *CHARK*, the response of the reporter genes to cytokinin was restored to a similar extent as in the *pAHK2:AHK2* line. In the *pAHK2-CHARK 25* line, the gene expression level of both *ARR5* and *ARR16* reached similar levels as in the wild type upon cytokinin induction (Fig. 7).

These results show that CHARK can restore in the cytokinin receptor mutants the response to cytokinin on the morphological and molecular level.

## Discussion

The *CHARK* gene of rice is an interesting case of an evolutionary event that illustrates the combination of different functional protein domains. However, the evolutionary origin of the *CHARK* sequence is currently unclear. The CHASE domain is most similar to the CHASE domains of the canonical cytokinin receptors from rice, and the Ser/Thr kinase domain shares the highest level of similarity with the kinase domains of lectins (Fig. 1). What could be the origin of this unusual domain combination? As there are no CHARK-type proteins (CHASE domain combined with Ser/Thr kinase domain) in any other organism, it seems most likely that this protein originated in the rice clade and thus was not introduced into rice by horizontal gene transfer. One way to combine two sequences from different gene loci is the process of *trans*-splicing (reviewed by^43,44^). The occurrence of *trans*-splicing has been documented in rice and in *Medicago sativa*^45,46^. While *trans*-splicing has rarely been reported, it might happen quite often. In fact, deep sequencing of the rice transcriptome revealed more than 200 sequences as product of *trans*-splicing with the vast majority combining mRNAs from different chromosomes^47^. However, *trans*-splicing would only explain how a chimeric mRNA is formed, not a chimeric gene. This can be accomplished by retro-transposition. The chimeric mRNA would be reverse transcribed into DNA and then integrated into the genome, creating a retrogene or retrocopy (reviewed by^48^). Retrogenes are found in animals and plants and depend on the activity of at least one of the different classes of retrotransposons. While the majority of retrogenes lack introns, a few acquired introns during evolution. One hallmark of retrogenes is the presence of flanking target site duplications or long terminal repeat sequences. However, such sequences are evolutionarily unstable and thus are bound to be found mainly in younger retrogenes^48^. The rice genome was found to contain 150 retrogenes most of which are expressed^49^. *CHARK* fulfills the criteria for a retrogene as it is surrounded by retrotransposon genes and repeat sequences typical for Miniature inverted repeat transposable elements (MITEs)^50^ (Supplemental Fig. S2). While the earliest diverging CHARK sequence (*O. longistaminata*) is intronless, some of the later diverging CHARKs seem to have acquired some introns (Fig. 2). However, as these introns are in different positions and are dispersed across different clades, they might have been acquired independently in a species-specific fashion.

In the past there have been only a few experiments characterizing the properties of CHARK. In contrast to other histidine kinases from rice, CHARK was not able to complement a histidine kinase mutation in the *SLN1* gene of yeast^21^. In a different study, overexpression of *CHARK* complemented the cytokinin sensitivity of root elongation in the *Arabidopsis cre1-1* mutant^39^. Here, we have shown that expression of *CHARK* under control of the *Arabidopsis AHK2* promoter complements several phenotypic aspects of the *ahh2 ahk3* mutant and conveys cytokinin sensitivity in *ahk2 ahk3* protoplast. Furthermore, it was shown that CHARK can bind cytokinin. Together this indicates that CHARK is an unusual but fully functional cytokinin receptor.

The fact that CHARK possesses a Ser/Thr kinase domain and not a His kinase domain as conventional cytokinin receptors do raises the question how it is complementing the *ahk2 ahk3* double mutant. There are several possible scenarios: (i) CHARK could directly interact with the histidine phosphotransfer proteins (HPts) and by phosphorylating them activate the canonical cytokinin signaling pathway; (ii) CHARK could heterodimerize with the AHKs and activate them upon binding of the cytokinin ligand; (iii) it could interact with type B response regulators or their downstream targets and thus trigger at least part of the cytokinin responses normally activated by the canonical signaling pathway or (iv) it could be part of a novel signaling pathway, that has some targets in common with the canonical cytokinin signaling pathway.

Of the different scenarios, the last one seems the least likely one. Different studies looking at the evolution of new signaling systems found that the most likely track for the establishment of a new pathway is a duplication of an existing one. In the course of evolution, ligand binding domains or DNA-target sites are modified or the new genes are expressed in a different temporal/spatial pattern with respect to the original pathway genes^4,51^. Sometimes these mechanisms are combined to rewire an existing mechanism to new inputs or functions^1^.

The third scenario, a posttranslational modification of response regulators or components further downstream of the TCS might be more plausible. While the different TCSs are the major part of the bacterial signaling systems, some Ser/Thr kinases have been identified in prokaryotes as well. These were shown to interact with response regulators or other components of the transcriptional machinery to modify their activity^52–54^. Such mechanisms could be envisioned by a phosphorylation of plant response regulators or downstream targets. While no Ser/Thr phosphorylation sites have been identified in response regulators, studies of the phosphoproteome have found numerous post-translational modifications of Ser/Thr residues in response to cytokinin treatment^55^.

The first two scenarios for the activity of CHARK postulate a direct activation of the cytokinin signaling pathway—either by interacting directly with the HPts or by heterodimerization with a classical cytokinin receptor. Both of these explanations are the most parsimonious, as they do not require additional factors or the evolution of novel phosphorylation sites. In a direct interaction with the HPts CHARK would directly substituting for the canonical cytokinin receptors. However, this raises the question how a Ser/Thr kinase could phosphorylate a HPT protein. The existence of kinases with dual activity, such as NTHK1 and NTHK2, which function as both His and as Ser/Thr kinases, might be a solution^56,57^. If CHARK would also have such a dual kinase activity, it could phosphorylate HPts from rice and thus trigger a cytokinin response. Such an interaction would most likely be conserved and thus it would be possible for CHARK to complement the cytokinin receptors from *Arabidopsis*. Consistently, addition of CHARK to a background of no cytokinin activity in *ahk2 ahk3* protoplasts was sufficient to activate the cytokinin signaling pathway. Alternatively, CHARK may be linked with the canonical cytokinin signaling pathway through a heterodimerization with a classical cytokinin receptor. Indeed, dimerization between different classical receptors have been shown before^58,59^. However, the last remaining cytokinin receptor in the *ahk2 ahk3* mutant, AHK4, is not expressed in leaf mesophyll cells^28^ but CHARK is active, making this an unlikely option.

Taken together, the data presented here demonstrate that CHARK is a novel type of receptor and that it is most likely the product of *trans*-splicing and retrotransposition. It can function as a cytokinin receptor, and CHARK complemented a canonical double receptor mutant of Arabidopsis on the molecular as well as on the morphological level. However, many questions need to be addressed, including its specific function(s) in rice, how precisely it interacts with the classical cytokinin pathway, and what its evolutionary trajectory is.

## Supplementary Information

Supplementary Information.
