# Supplementary material for: Characterization of CHARK, an unusual cytokinin receptor of rice
Source: Sci Rep. 2021 Jan 18;11:1722. doi: 10.1038/s41598-020-80223-2 (PMC7814049; doi:10.1038/s41598-020-80223-2)
Supplement: Supplementary file 1 — Supplementary Information. [file 41598_2020_80223_MOESM1_ESM.pdf]

Supplementary Information

for

Characterization of CHARK, an unusual cytokinin receptor of rice

Mhyeddeen Halawa, Anne Cortleven, Thomas Schmülling & Alexander Heyl

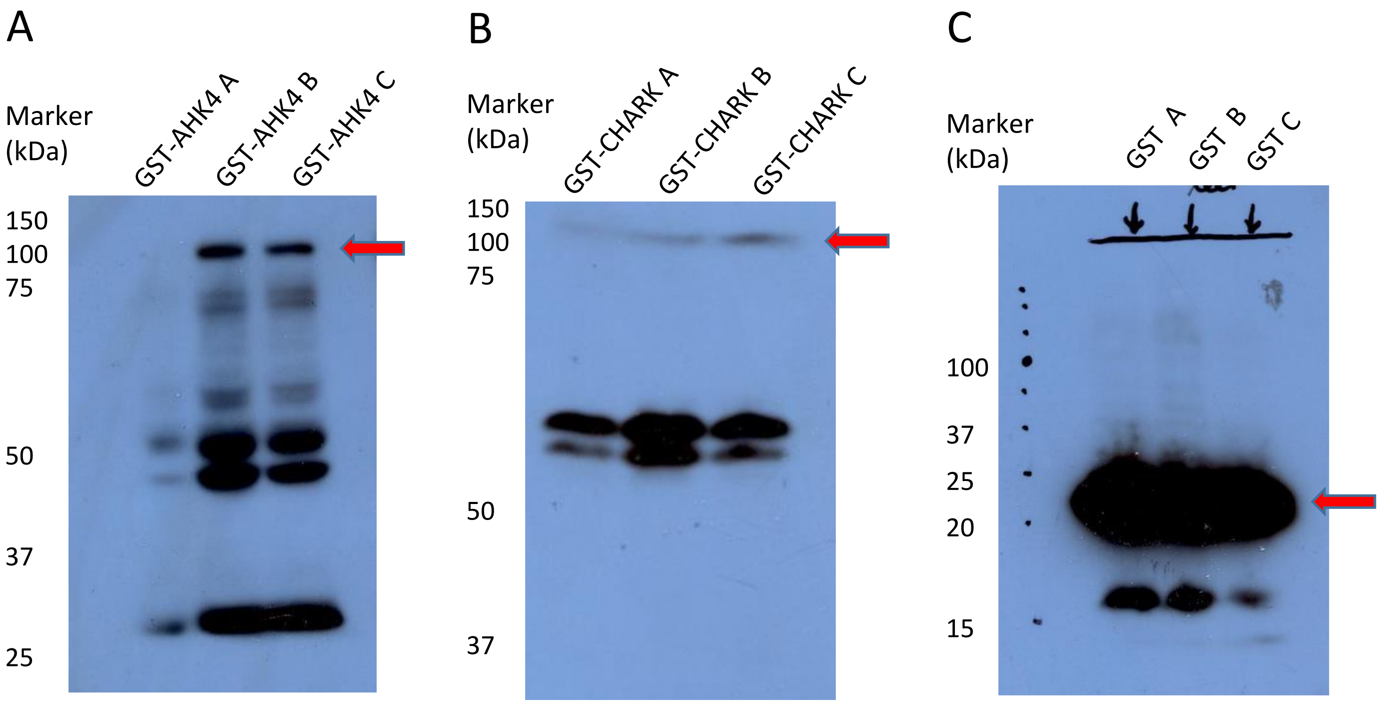

Supplemental Figure S1. Expression control of the GST fusion proteins in *E. coli*. Expression control of the fusion proteins in the three independent bacterial colonies which were examined in Figure 3. GST-containing bands were detected with a GST-specific antibody. The arrow marks the full-length protein, respectively (A, GST-AHK4; B, GST-CHARK; C, GST (empty vector)).

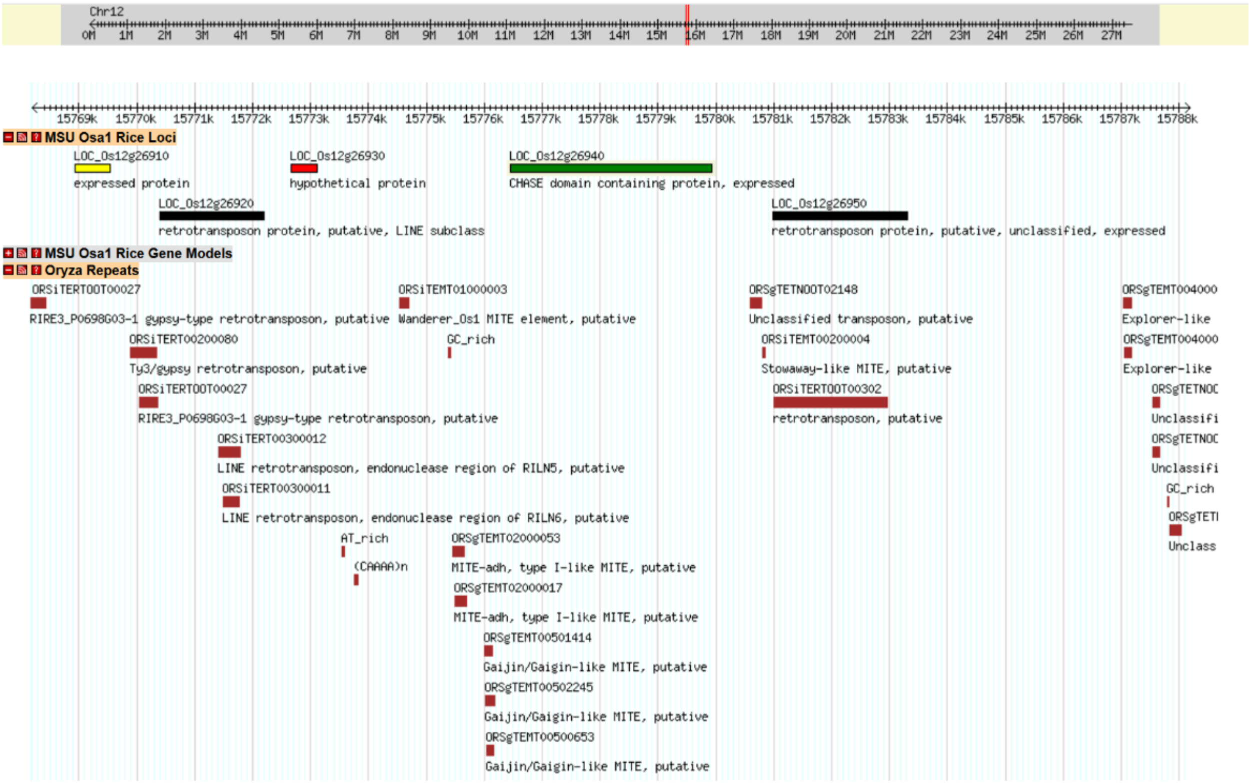

Supplemental Figure S2. Genomic environment of the *CHARK* gene. The genomic environment of CHARK was analyzed using the genomic browser at the Rice Genome Annotation Project database (rice.plantbiology.msu.edu).

Table S1: Primers sequences used in this study

Quantitative real-time PCR primers

Gene Name	ATG number	Forward primer	Reverse primer
UBC21	At5g25760	ACTCTTAGCCAAGTAGTGCTCC	GAATCACGGCCAACAATC
EF1A	At5g60390	TGAGCACGCTCTTCTTGCTTTCA	GGTGGTGGCATCCATCTTGTTACA
CHARK	Os12t0454800-01	TGAGGGAGTTCAAGGCAGAG	GGTGGAGGTAGAGCAAAGCA
ARR5	At3g48100	CTACTCGCAGCTAAAACGC	GCCGAAAGAATCAGGACA
ARR16	AT2G40670	TCAGGAGGTTCTTGTTCGTCTT	AACCCAAATACTCCAATGC
